# Perceptions of asymptomatic malaria infection and their implications for malaria control and elimination in Laos

**DOI:** 10.1371/journal.pone.0208912

**Published:** 2018-12-11

**Authors:** Bipin Adhikari, Koukeo Phommasone, Tiengkham Pongvongsa, Xayaphone Soundala, Palingnaphone Koummarasy, Gisela Henriques, Thomas J. Peto, Lorenz von Seidlein, Nicholas J. White, Nicholas P. J. Day, Arjen M. Dondorp, Paul N. Newton, Phaik Yeong Cheah, Mayfong Mayxay, Christopher Pell

**Affiliations:** 1 Mahidol-Oxford Tropical Medicine Research Unit, Faculty of Tropical Medicine, Mahidol University, Bangkok, Thailand; 2 Centre for Tropical Medicine and Global Health, Nuffield Department of Medicine, Churchill Hospital, Oxford, United Kingdom; 3 Kellogg College, University of Oxford, Oxford, United Kingdom; 4 Lao-Oxford-Mahosot Hospital-Wellcome Trust Research Unit (LOMWRU), Microbiology Laboratory, Mahosot Hospital, Vientiane, Laos; 5 Faculty of Tropical Medicine, Mahidol University, Bangkok, Thailand; 6 Savannakhet Provincial Health Department, Savannakhet Province, Laos; 7 Department of Life Science, Imperial College London, London, United Kingdom; 8 The Ethox Centre, Nuffield Department of Population Health, University of Oxford, Oxford, United Kingdom; 9 Institute of Research and Educational Development, University of Health Sciences, Vientiane, Laos; 10 Centre for Social Science and Global Health, University of Amsterdam, Amsterdam, The Netherlands; 11 Amsterdam Institute for Global Health and Development, Amsterdam, The Netherlands; Institut de recherche pour le developpement, FRANCE

## Abstract

**Background:**

In the Greater Mekong Sub-region (GMS), malaria elimination efforts are targeting the asymptomatic parasite reservoirs. Understanding community perceptions about asymptomatic malaria infections and interventions that target this reservoir is critical to the design of community engagement. This article examines knowledge, attitudes, perceptions and practices related to asymptomatic malaria infections and mass drug administration (MDA) in malaria-endemic villages in southern Savannakhet Province, Laos.

**Methods:**

A questionnaire consisting of questions on socio-demographic characteristics, knowledge, attitudes, perceptions and practices on malaria and MDA was administered to each household head or representative (n = 281) in four villages. These topics were also further discussed in 12 single-gender focus group discussions (FGDs). The FGDs were conducted in all four villages and consisted of eight to 10 participants.

**Results:**

A minority (14.2%; 40/281) of respondents agreed that a seemingly healthy person could have malaria parasite in his or her blood. Half (52%; 146/281) disagreed and one third (33.8%, 95/281) were unsure. Respondents who responded that “MDA aims to cure everyone” [AOR = 4.6; CI: 1.6–13.1], “MDA is to make our community malaria free” [AOR = 3.3; CI: 1.3–8.1] and “I will take part in future MDA” [AOR = 9.9; CI: 1.2–78.8] were more likely to accept the idea of asymptomatic malaria. During FGDs, respondents recalled signs and symptoms of malaria (fever, chills and headache), and described malaria as a major health problem. Symptomatic and asymptomatic malaria infections were associated with their work in the forest and living conditions. Measures described to eliminate malaria included using mosquito nets, wearing long-sleeved clothes and taking medicine when symptomatic. Most respondents were unaware of MDA as a tool to eliminate malaria.

**Conclusions:**

Awareness of asymptomatic malaria infections, and MDA as a tool to eliminate malaria, was low. With the need to target asymptomatic malaria carriers for elimination efforts in the GMS, as well as informing target groups about asymptomatic infection, accompanying community engagement must build trust in interventions through the active collaboration of government stakeholders, key local persons and community members. This entails training and devolving responsibilities to the community members to implement and sustain the control and elimination efforts.

## Introduction

The emergence of antimalarial resistance in the Greater Mekong sub-Region (GMS) has triggered greater efforts to contain its spread within the region and beyond [1, 2]. This, together with general declines in malaria incidence, has prompted malaria control programs in the region to set targets for the elimination of *falciparum* malaria by 2025 and all malaria species by 2030 [3]. The Lao People’s Democratic Republic (Laos) has adopted the goal of eliminating malaria by 2025 [4].

Achieving such goals is challenging for several reasons. First, the emerging resistance to all anti-malarials poses a serious challenge for containment [5]. Second, malaria tends to cluster in populations that live along international borders, within forested areas and in forest fringes [3]. These communities are often geographically isolated and highly mobile, have low levels of literacy, experience language barriers, include ethnic minorities, endure a lack of health care services and experience high levels of human-mosquito interaction [6, 7].

With recent declines in clinical malaria, there is increasing evidence that asymptomatic parasite carriers play a critical role in sustaining transmission [2, 8–11]. Seemingly healthy individuals can be chronic carriers of asexual and sexual (gametocytes) forms of the parasite that are critical for transmission [10, 12]. This is particularly the case in low- and seasonal-transmission areas where people with chronic, asymptomatic infections may serve as the infectious reservoir for transmission in subsequent seasons [12]. Data from Cameroon, The Gambia, Mali and Senegal indicate that over 25% of individuals with sub-microscopic gametocytes were capable of infecting mosquitoes [13]. A recent study in Laos found 20% (175/888) of the seemingly healthy individuals were infected with *Plasmodium* species [4].

Elimination programs must therefore target asymptomatic malaria reservoirs to eliminate rapidly the disease. Mass drug administration (MDA), whereby a complete dose of antimalarial treatment is provided to the entire targeted population, regardless of infection status, is one such approach [2, 14, 15]. In the absence of more promising interventions, the threat of a public health emergency if multidrug resistant malaria reaches Africa has prompted scientists to consider MDA as an approach to accelerate elimination in addition to other malaria prevention and control [2, 16]. Together with the ethical justification for such an approach [15, 16], recently scaled up MDA was found effective in Thai-Myanmar border [17]. Including MDA, strategies such as mass screening and treatment (MSAT), focal screening and treatment (FSAT) and voluntary screening and testing (VSAT) for malaria require testing all people in a geographical area and treating only positive cases [18, 19]. All of these approaches entail people taking anti-malarials when asymptomatic.

Recent MDAs for targeted falciparum malaria elimination have highlighted the challenges related to convincing healthy people to take anti-malarials [6, 20–23]. The rationale for this approach involves complex biological concepts and communicating them to people with low levels of formal education and frequently across language barriers [6, 20, 24]. Furthermore, local declines in clinical *falciparum* cases might mean that malaria is no longer a priority in target communities and reduce the willingness of some people to take anti-malarials [21].

The situation is further complicated by the presence of *P*. *vivax* and *P*. *falciparum* in South East Asia [25] and *P*. *vivax* has been found to dominate the overall prevalence of plasmodium infections [4, 9, 10, 26]. This complex *P*. *vivax* parasite has a latent liver stage, hypnozoites, which can cause relapsing infections for months and years [4, 26–28]. *P*. *vivax* persistence can sustain a parasite reservoir for ongoing transmission and radical therapy targeting this parasite requires an addition of a 14 days course of primaquine [28], which carries the risk of hemolysis in G6PD-deficient individuals [10, 29, 30]. In remote communities of the GMS, knowledge about malaria species and the mechanisms of infection is however low [6, 21, 24]. Yet after the elimination of *P*. *falciparum* infections *P*. *vivax* infections are likely to persist.

To date, three articles have reported malaria-related knowledge, attitudes and practices in Laos [31–33]. However, none have explored perceptions of asymptomatic malaria infections in rural malaria endemic areas.

This article draws on mixed-methods formative research conducted prior to a pilot study of MDA for targeted malaria elimination (TME) in Nong District, southern Savannakhet Province, Laos. The article explores understandings of asymptomatic malaria infections in a geographically remote area, with a view to designing community engagement strategies to accompany interventions that target the parasite reservoir (such as MDA, MSAT and VSAT).

## Materials and methods

### Study context

Four villages in Nong District were chosen for the TME study on the malaria prevalence survey conducted during June and July 2015 in Thappangthong and Nong District, Savannakhet Province [4]. All four villages; Oi Tan Tip (OTP), Phoun Mak Mee (PMM), Tha The (TT) and Xuang Tai (XT) are home to members of ethnic minority groups (*Mangkong* = 200; 71.2%, *Tree* = 64; 22.8%, and *Phu Thai* = 6). Oi Tan Tip is farthest (≥50km) from the Nong District town, lying close to the Vietnam border, and Xuang Tai was closest (≥10 km). The other villages, PMM and TT are about 25 km and 15 km away from the district town respectively.

Residents of all four villages primarily rely on subsistence farming, including migratory, swidden cultivation of staple foods, such as sticky rice, maize, and cash crops such as cassava. Besides staple food, community members visit local forests to collect foods, such as bamboo shoots, wild rodents, beetles and other insects. Most community members reared domestic animals, such as cows, pigs, chicken, buffaloes and goats, which are also their source of cash. For instance, when in urgent need of money, community members sell these animals to Vietnamese mobile traders for cash.

There are limited health facilities in the study villages. In PMM, one health center is located within the settlement, but the residents of the other villages had to travel from 5km (for TT) to over (10km for OTP) to reach the nearest health center.

### Study participants and questionnaire

Based on the census conducted for TME study, a total of 281 households (OTP = 65, PMM = 74, TT = 82 and XT = 60) were included in this survey. A questionnaire was administered to one household member above the age of 18 years, preferably household head from all households in the four study villages. Two Laotian social scientists interviewed respondents at their household following written informed consent. If representatives of the household were not present during the survey, they were followed up during the consecutive days. None of the household representative refused to participate. Respondents representing a total of two households were away and interviewed once they returned. Each interview lasted for 30 to 45 minutes.

Each questionnaire was divided into five parts: 1) general information about the interview, interviewer and interviewee; 2) socio-demographic characteristics, such as age, sex, religion and marital status; 3) health seeking behavior; 4) malaria-related knowledge, attitudes and practices; 5) knowledge and attitudes toward MDA (S1 Questionnaire). Based on the research question of this study, all sections except section 3 submitted as manuscript elsewhere are included. Variables in the questionnaire were designed after carefully reviewing the past studies from Laos [34–36] and elsewhere [37–41]. The questionnaire was designed in English, then translated into Laotian and back translated into English to ensure validity.

The questionnaires were pre-tested with members of the study team and 15 volunteer participants around Mahosot Hospital in Vientiane. Further pre-testing was carried out in Nong District in six households. Any ambiguity found was corrected and simplified. After the questionnaire was finalized, it was entered into the Open Data Kit (ODK; Available online: https://opendatakit.org/) application on a smartphone.

### Data management and analysis

All data entered into ODK were extracted into an Excel sheet (Microsoft Excel 2013) and analyzed in IBM SPSS Statistics for Windows, Version 24.0, Armonk, NY: IBM Corp.

Each respondent was asked whether a healthy looking person could have malaria parasite in his/her blood. The response to this question was treated as an outcome variable and were further dichotomized into “Yes” and “No” to explore the associations with respondents’ awareness of the possibility of asymptomatic malaria. Following a descriptive analysis (frequency and percentage), the data were analysed using Chi squared and Fisher’s exact test as appropriate to determine associations. All variables (socio-demographic, knowledge, attitude, practice and perceptions on malaria and MDA) were tested for association with the outcome variable and an association was considered significant at p value ≤0.05.

Further analysis using logistic regression was carried out in which variables were selected based on the significant association with the perception on asymptomatic malaria infections and the relevance to the research question. For the logistic regression analysis, thus selected variables were dichotomized into two categories; with presence of certain characters/conditions = 1 and absence = 0. First, a univariate analysis was conducted with the outcome variable (perception on asymptomatic malaria infection categorized as “Yes” = 1 and “No” = 0) to calculate crude odds ratios. In the multi-variate analysis, selected variables were included into the model to calculate the adjusted odds ratio. Statistical significance was considered if p value ≤0.05.

### Focus group discussions

To explore further the issues addressed in the questionnaire, a series of focus group discussions were also undertaken in all four study villages. In total, 12 FGDs were conducted with 100 participants from the four villages and their sub-villages. Each FGD involved eight to 10 participants, selected by simple randomization (lottery method) during village meetings. Aware of the tendency for conformism, and patriarchy in the study communities [20, 24], to encourage the active participation of female respondents, discussions were conducted in single-sex groups by a male or female social scientist. Based on a joint decision among participants, the discussions took place at one of their houses.

An open-ended questionnaire guide was used to lead the discussion and each FGD lasted for about 30 to 45 minutes. The FGDs were held in *Pasha Lao* and the local language with translation provided by a TME staff member or bilingual volunteer from the village. People gave informed oral consent at the community meeting and each selected participant provided individual consent.

All FGDs were audio-recorded and transcribed and translated into English by the FGD facilitators. Translated and transcribed FGD transcripts were analyzed using qualitative data analysis software (QSR NVivo 11). All transcripts were coded line by line using pre-set themes (deductive approach) as nodes including addition of nodes for emerging themes (inductive approach). Based on the research question, all the nodes were analyzed for the pattern and the content and are presented in the manuscript below.

### Ethics approval and consent to participate

Ethical approval for the study was received from the Lao National Ethics Committee for Health Research (Ref. No. 013-2015/NECHR), Government of the Lao PDR and the Oxford Tropical Research Ethics Committee (1015–13). Written informed consent were sought from each participant before each interview and FGD.

## Results

### Questionnaire-based survey

#### Socio-demographic characteristics and awareness of asymptomatic malaria infections

Most respondents were aged below 50 years (mean age = 38.9 years; 229/281; 81.5%), male (201/281; 71.5%), household head (188/281; 66.9%), from the ethnic minorities (269/281; 95.7%), identified as animist (272/281; 96.8%), illiterate (214/281; 76.2%), rice farmers (254/281; 90.4%) with a monthly income (192/281; 68.3%) of less than 500,000 Kip (60 USD).

Of the 281 participants, only 40 (14.2%) reported that a seemingly healthy person could have malaria parasite in his/her blood. Over half (146/281; 52%) rejected this idea and a third (95/281; 33.8%) responded that they did not know (Table 1 and S1 Tables).

**Table 1:** Socio-demographic characteristics of participants in relation to perception on asymptomatic malaria infections (n = 281).

Characteristics	Total	Possibility of asymptomatic malaria infections		p-value
	Number (%)	Yes (n = 40)	No (n = 241)	
**Village**				
OTP	65 (23.1)	16 (40)	49 (20.3)	**<0.001**
PMM	74 (26.3)	2 (5)	72 (29.9)	
TT	82 (29.2)	5 (12.5)	77 (32)	
XT	60 (21.4)	17 (42.5)	43 (17.8)	
**Respondent status**				
Family head	188 (66.9)	29 (72.5)	159 (66)	0.35
Wife of family head	70 (24.9)	10 (25)	60 (24.9)	
Other	23 (8.2)	1 (2.5)	22 (9.1)	
**Age Group**				
≤30 years	91 (32.4)	13 (32.5)	78 (32.4)	0.28
31–50 years	138 (49.1)	23 (57.5)	115 (47.7)	
≥51 years	52 (18.5)	4 (10)	48 (19.9)	
Mean = 38.9±14.5, min = 18 and max = 100				
**Sex**				
Male	201 (71.5)	30 (75)	171 (71)	0.37
Female	80 (28.5)	10 (25)	70 (29)	
Ethnicity*				
Lao Theung	269 (95.7)	32 (80)	237 (98.3)	**<0.001**
Other	12 (4.3)	8 (20)	4 (1.7)	
**Religion**				
Buddhist	9 (3.2)	8 (20)	1 (0.4)	**<0.001**
Animist	272 (96.8)	32 (80)	240 (99.6)	
**Marital Status**				
In relationship	262 (93.2)	39 (97.5)	223 (92.5)	0.21
Not in relationship	19 (6.8)	1 (2.5)	18 (7.5)	
**Literacy**				
Literate	67 (23.8)	18 (45)	49 (20.3)	**0.001**
Illiterate	214 (76.2)	22 (55)	192 (79.7)	
**Education in years**				
Not attended School	204 (72.6)	22 (55)	182 (75.5)	**0.008**
Attended School	77 (27.4)	18 (45)	59 (24.5)	
**Occupation**				
Farmer	254 (90.4)	29 (72.5)	225 (93.4)	**<0.001**
Other	27 (9.6)	11 (27.5)	16 (6.6)	
**Monthly Income**				
≤500,000 kip	192 (68.3)	14 (35)	178 (73.9)	**<0.001**
500,001 to 2,000,000 kip	41 (14.6)	11 (27.5)	30 (12.4)	
≥ 2000,001	27 (9.6)	11 (27.5)	16 (6.6)	
Don't know	21 (7.5)	4 (10)	17 (7.1)	
**Do you have toilet facility at home?**				
Yes	22 (7.8)	4 (10)	18 (7.5)	0.38
No	259 (92.2)	36 (90)	223 (92.5)	
**Did you migrate from any other village?**				
Yes	61 (21.7)	13 (32.5)	48 (19.9)	0.06
No	220 (78.3)	27 (67.5)	193 (80.1)	
**How far is the forest from your house in km?**				
≤1 km	97 (34.5)	10 (25)	87 (36.1)	0.24
1.1 to 2 km	76 (27)	13 (32.5)	63 (26.1)	
≥2.1 km	49 (17.4)	5 (12.5)	44 (18.3)	
NA	59 (21)	12 (30)	47 (19.5)	
**How often do you go to forest?**				
Everyday	171 (60.9)	24 (60)	147 (61)	0.26
Every alternate day	68 (24.2)	7 (17.5)	61 (25.3)	
≥Weekly	42 (14.9)	9 (22.5)	33 (13.7)	

*Ka Tarng = 1 (0.4%), Lao Loum = 3 (1.1%), Mangkong = 200 (71.2), Phu Thai = 6 (2.1%), Ta Oi = 3 (1.1%), Tree = 64 (22.8%), Vietnamese = 4 (1.4%)

Respondents’ literacy (illiterate: 22/214; 10.3% versus literate: 18/67; 26.9%; p = 0.001), education status (not attended school: 22/204; 10.8% versus attended school: 18/77; 23.4%; p = 0.008), occupation (farmer: 29/254; 11.41% versus non-farmer: 11/27;40.7; <0.001), monthly income (lowest income group: ≤500,001 kip: 14/192; 7.3% versus middle income group: 500,001 to 2,000,000 kip: 11/41; 26.8%; p<0.001), influenced their response about the possibility of asymptomatic malaria infection.

#### Malaria-related knowledge, practices and perceptions

Almost all respondents, 260/281 (92.5%) had heard about malaria before (Tables 2 and 3 and S1 Tables). Among these 260 respondents, most could recall the symptoms of malaria such as fever: 231/260; 88.8%, headache: 190/260; 73.1% and chills: 227/281; 87.3%. In the past, the majority received information on malaria from health workers (193/281; 74.2%) and knew that malaria was transmitted by mosquitoes (230/281; 81.9%). Respondents who recalled that mosquitoes transmit malaria (37/230; 16.1%; compared to those who did not: 3/51; 5.9%; p = 0.004) were likely to report the possibility of asymptomatic malaria infection. The majority of respondents recalled that using a mosquito net at home (274/281; 97.5%) and wearing long sleeves in the forest (262/281; 93.2%) can prevent mosquito bites. Also, the majority (256/281; 91.1%) recalled sleeping under the mosquito net the previous night at home. On further prompting, most respondents described malaria as a deadly disease (265/281; 94.3%) and were confident that malaria was cured by medicine (275/281; 97.9%).

**Table 2:** Knowledge, practices, perceptions and attitudes towards malaria of participants in relation to perception on asymptomatic malaria infections (n = 281).

Characteristics	Total	Possibility of asymptomatic malaria infections		p-value
	Number (%)	Yes (n = 40)	No (n = 241)	
**Have you heard of malaria before?**				
Yes	260 (92.5)	38 (95)	222 (92.1)	0.71
No	18 (6.4)	2 (5)	16 (6.6)	
Don't know	3 (1.1)	0	3 (1.2)	
What are the symptoms of malaria? (n = 260)*				
Fever	231 (88.8)	33 (86.8)	198 (89.2)	0.42
Headache	190 (73.1)	32 (84.2)	158 (71.2)	0.06
Muscle pain	46 (17.7)	12 (31.6)	34 (15.3)	**0.01**
Vomiting	45 (17.3)	7 (18.4)	38 (17.1)	0.5
Chills	227 (87.3)	32 (84.2)	195 (87.8)	0.34
Where did you get the information on malaria from? (n = 260)*				
Village Meetings	3 (1.2)	1 (2.6)	2 (0.9)	0.39
Health workers	193 (74.2)	24 (63.2)	169 (76.1)	0.07
Banners	2 (0.8)	2 (5.3)	0	**0.02**
Other	49 (18.8)	8 (21.1)	41 (18.5)	0.42
Don’t know	41 (15.8)	13 (34.2)	28 (12.6)	**0.002**
Where would you like to get health information from?*				
Village Meetings	59 (21)	9 (22.5)	50 (20.7)	0.47
Health workers	137 (48.8)	23 (57.5)	114 (47.3)	0.15
Banners	56 (19.9)	6 (15)	50 (20.7)	0.27
Entertainment	52 (18.5)	9 (22.5)	43 (17.8)	0.3
Other	13 (4.6)	2 (5)	11 (4.6)	0.57
Don’t know	31 (11)	5 (12.5)	26 (10.8)	0.46
Malaria is transmitted from*				
Water	14 (5)	2 (5)	12 (5)	0.61
Soil	1 (0.4)	1 (2.5)	0	0.14
Forest	55 (19.6)	9 (22.5)	46 (19.1)	0.37
Mosquito	230 (81.9)	37 (92.5)	193 (80.1)	**0.04**
God	1 (0.4)	0	1 (0.4)	0.85
Uncleaned Surrounding	30 (10.7)	4 (10)	26 (10.8)	0.57
Don’t know	29 (10.3)	0	29 (12)	**0.009**
How do you prevent mosquito bites at home?*				
Using mosquito net	274 (97.5)	40 (100)	234 (97.1)	0.33
Using repellants	5 (1.8)	1 (2.5)	4 (1.7)	0.53
By smoking	2 (0.7)	0	2 (0.8)	0.73
Wearing Sleeves	16 (5.7)	3 (7.5)	13 (5.4)	0.4
How do you prevent mosquito bites at forest?*				
Using mosquito net	1 (0.4)	0	1 (0.4)	0.85
Burning fire	11 (3.9)	2 (5)	9 (3.7)	0.48
Using repellants	7 (2.5)	5 (12.5)	2 (0.8)	**0.001**
By smoking	1 (0.4)	1 (2.5)	0	0.14
Wearing Sleeves	262 (93.2)	39 (97.5)	223 (92.5)	0.21

*Multiple answers were possible and percentage does not add to 100, analyses were made against "yes" and "no".

**Table 3:** Knowledge, practices, perceptions and attitudes towards malaria of participants in relation to perception on asymptomatic malaria infections (n = 281).

Characteristics	Total	Possibility of asymptomatic malaria infections		p-value
	Number (%)	Yes (n = 40)	No (n = 241)	
**Did you sleep under the mosquito net last night?**				
Yes	256 (91.1)	38 (95)	218 (90.5)	0.27
No	25 (8.9)	2 (5)	23 (9.5)	
**If Yes, how often do you sleep under the mosquito net?**				
Everyday	247 (96.5)	35 (92.1)	212 (97.2)	0.13
Sometimes (>2–3 days in a week)	9 (3.5)	3 (7.9)	6 (2.8)	
**How many mosquito nets do you have at your home?**				
≤2	156 (55.5)	23 (57.5)	133 (55.2)	0.46
≥3	125 (44.5)	17 (42.5)	108 (44.8)	
**Do you think you can get malaria if anybody in your family/neighbor has malaria?**				
Yes	164 (58.4)	27 (67.5)	137 (56.8)	0.26
No	83 (29.5)	11 (27.5)	72 (29.9)	
Don't know	34 (12.1)	2 (5)	32 (13.3)	
If Yes, how? (n = 164)*				
Touch	75 (45.7)	8 (29.6)	67 (48.9)	**0.05**
Air	11 (6.7)	0	11 (8)	0.12
Mosquito	108 (65.9)	21 (77.8)	87 (63.5)	0.11
Water	33 (20.1)	4 (14.8)	29 (21.2)	0.32
Food	34 (20.7)	4 (14.8)	30 (21.9)	0.29
How do we know if a person has malaria?*				
Blood	178 (63.3)	30 (75)	148 (61.4)	0.06
Fever	73 (26)	14 (35)	59 (24.5)	0.11
Health worker	90 (32)	11 (27.5)	79 (32.8)	0.32
Other	33 (11.7)	2 (5)	31 (12.9)	0.11
**Is malaria a deadly disease?**				
Yes	265 (94.3)	40 (100)	225 (93.4)	0.24
No	12 (4.3)	0	12 (5)	
Don't know	4 (1.4)	0	4 (1.7)	
**Are you scared of malaria?**				
Yes	277 (98.6)	39 (97.5)	238 (98.8)	0.58
No	3 (1.1)	1 (2.5)	2 (0.4)	
Don't know	1 (0.4)	0	1 (0.4)	
**Can malaria be cured by medicine?**				
Yes	275 (97.9)	38 (95)	237 (98.3)	0.4
No	3 (1.1)	1 (2.5)	2 (0.8)	
Don't know	3 (1.1)	1 (2.5)	2 (0.8)	

*Multiple answers were possible and percentage does not add to 100, analyses were made against "yes" and "no".

#### Knowledge, perceptions and attitudes towards MDA

Most respondents described malaria as an important problem in their communities (178/281; 63.3%) (Table 4 and S1 Tables). Respondents who agreed with the idea of asymptomatic malaria infections also agreed that a healthy person with malaria parasites in his or her blood can transmit the infection (23/103; 22.3%; p<0.001). When asked if medicine should be provided to community members with asymptomatic malaria infections, three-quarters (78/103) answered “yes”, and this was to “to cure all villagers” (11/29; 37.9%). The majority (241/281; 86%) had not heard about the malaria elimination programme in their village. More than half (162/281; 57.7%) stated that malaria can be eliminated from their village and with regard to ways (options) to eliminate the disease, they two thirds selected giving medicine to all the villagers (105/162; 64.8%), half chose using mosquito nets (81/162; 50%) and a sixth opted for cleaning the surroundings (23/162; 14.2%). The majority of respondents who thought that malaria elimination was possible were more likely to recognize the possibility (Yes: 30/162; 18.5% versus No and Don’t know: 10/109; 9.1%; p = 0.08) of asymptomatic malaria infections. Most respondents showed enthusiasm (208/281; 74%) for participation in future malaria elimination campaign as a volunteer and were likely to recognize (Yes: 35/208; 16.8% versus No: 5/60; 8.3%; p = 0.08) the possibility of asymptomatic malaria infections.

**Table 4:** Knowledge, perceptions and attitudes towards MDA of participants in relation to perception on asymptomatic malaria infections (n = 281).

Characteristics	Total	Possibility of asymptomatic malaria infections		p-value
	Number (%)	Yes (n = 40)	No (n = 241)	
**Is malaria a big problem in your community?**				
Yes	178 (63.3)	29 (72.5)	149 (61.8)	0.06
No	43 (15.3)	8 (20)	35 (14.5)	
Don't know	60 (21.4)	3 (7.5)	57 (23.7)	
**Do you think a person in your village can have malaria parasite without being sick?**				
Yes	34 (12.1)	26 (65)	8 (3.3)	**<0.001**
No	144 (51.2)	9 (22.5)	135 (56)	
Don't know	103 (36.7)	5 (12.5)	98 (40.7)	
**Can a healthy person with malaria parasite in his body transmit to others?**				
Yes	103 (36.7)	23 (57.5)	80 (33.2)	**<0.001**
No	94 (33.5)	13 (32.5)	81 (33.6)	
Don't know	84 (29.9)	4 (10)	80 (33.2)	
**If Yes, should we provide medicine to all the villagers?**				
Yes	78 (75.7)	17 (73.9)	61 (76.3)	0.26
No	19 (18.4)	6 (26.1)	13 (16.3)	
Don't know	6 (5.8)	0	6 (7.5)	
If Yes, why? (n = 78)*				
To cure all villagers	29 (37.2)	11 (64.7)	18 (29.5)	**0.01**
To eliminate malaria from the village	15 (19.2)	4 (23.5)	11 (18)	0.42
To prevent malaria transmission in the village	41 (52.6)	9 (52.9)	32 (52.5)	0.59
To prevent us from malaria	34 (43.6)	7 (41.2)	27 (44.3)	0.52
**Have you heard of malaria elimination in your village?**				
Yes	40 (14.2)	8 (20)	32 (13.3)	0.14
No	224 (79.7)	32 (80)	192 (79.7)	
Don't know	17 (6)	0	17 (7.1)	
**Do you think malaria can be eliminated from your village?**				
Yes	162 (57.7)	30 (75)	132 (54.8)	**0.04**
No	66 (23.5)	7 (17.5)	59 (24.5)	
Don't know	53 (18.9)	3 (7.5)	50 (20.7)	
If Yes, how? (n = 162)*				
By giving medicine to all villagers	105 (64.8)	24 (80)	81 (61.4)	**0.04**
By using mosquito net	81 (50)	17 (56.7)	64 (48.5)	0.27
By taking regular medicine	9 (5.6)	1 (3.3)	8 (6.1)	0.47
By cleaning surrounding	23 (14.2)	6 (20)	17 (12.9)	0.23
Don't know	11 (6.8)	1 (3.3)	10 (7.6)	0.35
Other	30 (18.5)	6 (20)	24 (18.2)	0.49
**Would you participate in malaria elimination as a volunteer?**				
Yes	208 (74)	35 (87.5)	173 (71.8)	**0.08**
No	60 (21.4)	5 (12.5)	55 (22.8)	
Don't know	13 (4.6)	0	13 (5.4)	
If Yes, why? (n = 208)*				
I want to make my community free from malaria	127 (61.1)	29 (82.9)	98 (56.6)	**0.002**
I want to help my community	85 (40.9)	14 (40)	71 (41)	0.53
Malaria is a big problem in my community	6 (2.9)	2 (5.7)	4 (2.3)	0.26
Other	14 (6.7)	0	14 (8.1)	0.06
Don't know	21 (10.1)	1 (2.9)	20 (11.6)	0.09
**Have your ever heard about MDA before?**				
Yes	257 (91.5)	36 (90)	221 (91.7)	0.66
No	16 (5.7)	2 (5)	14 (5.8)	
Don't know	8 (2.8)	2 (5)	6 (2.5)	
**Would you take part in MDA in future?**				
Yes	198 (70.5)	39 (97.5)	159 (66)	**<0.001**
No	82 (29.2)	1 (2.5)	81 (33.6)	
Don't know	1 (0.4)	0	1 (0.4)	

*Multiple answers were possible and percentage does not add to 100, analyses were made against "yes" and "no".

#### Multivariate logistic regression

Using the logistic regression model, the following factors were associated with recognizing the possibility of asymptomatic malaria infections (Table 5). 1. Interest in taking part in future MDAs (AOR = 9.9; CI = 1.2 to 78.8; p = 0.02). Respondents were asked if MDA for malaria was going to happen in their village in future, would they take part. Those who responded yes were likely to recognize the possibility of asymptomatic infection. 2. Understanding that the rationale of MDA is to cure all people, including those with asymptomatic infections (AOR = 4.6; CI = 1.6 to 13.1; p = 0.004). Respondents who responded that providing antimalarials to all community members (was to cure all people with malaria and asymptomatic infections) were likely to recognize the possibility of asymptomatic malaria infection 3. Respondents who indicated a desire to participate in MDA as a volunteer (assistant) (AOR = 3.3; CI: 1.3 to 8.1; p = 0.008). Respondents motivated to contribute as a volunteer in MDA were more likely to accept the possibility of asymptomatic malaria infection.

**Table 5:** Logistic regression on knowledge, attitude and perceptions predicting the perception on asymptomatic malaria infections.

Covariates and analyzed sample	Total	Univariate Analysis	Multivariate analysis
	Number (%)	Crude OR (95% CI)	AOR* (95% CI)
Animist religion (n = 281)	272 (96.8)	0.01 (0.002–0.13)	0.09 (0.001–0.14)
Living for less than 10 years in the village (n = 61)	28 (45.9)	6.0 (2.58–14.03)	2.85 (0.79–10.19)
Forest less than 1 km away (n = 281)	97 (34.5)	0.59 (0.27–1.26)	0.19 (0.04–0.9)
MDA is to cure all people (n = 78)	29 (37.2)	4.69 (2.0–10.92)	4.66 (1.65–13.15)
I want to make my community free from malaria (n = 208)	127 (61.1)	3.84 (1.83–8.0)	3.35 (1.36–8.15)
I will take part in MDA in future (n = 281)	198 (70.5)	20.11 (2.71–149.0)	9.97 (1.26–78.85)

*AOR = Adjusted Odds Ratio; adjusted with age and sex

### Focus group discussions

#### Characteristics of the participants

One hundred community members were chosen at random from the four study villages and their sub-villages to participate in six gender-specific FGDs (Table 6). The mean age of respondents was 37 years, ranging from 18 to 80 years. Fifty two of the 100 respondents were male. All participants were farmers and the majority (82/100) had not attended school.

**Table 6:** Socio-demographic characteristics of FGD Participants (n = 100).

Characteristics		Village: Number (%)					
	Total	Oi	Tantip	PMM	Keng and Appok	TT main	XT
**Age Group**							
≤35 years	51 (51)	7 (13.7)	12 (23.5)	6 (11.8)	13 (25.5)	7 (13.7)	6 (11.8)
>36 years	49 (49)	9 (18.4)	4 (8.2)	12 (24.5)	5 (10.2)	9 (18.4)	10 (20.4)
Mean = 37.58±12.71; Range = 18–80 years							
**Sex**							
Male	52 (52)	8 (15.4)	8 (15.4)	10 (19.2)	10 (19.2)	8 (15.4)	8 (15.4)
Female	48 (48)	8 (16.7)	8 (16.7)	8 (16.7)	8 (16.7)	8 (16.7)	8 (16.7)
**Occupation**							
Farmer	100 (100)	16 (16)	16 (16)	18 (18)	18 (18)	18 (18)	16 (16)
**Education Groups**							
Not attended school	82 (82)	12 (14.6)	10 (12.2)	13 (15.9)	17 (20.7)	14 (17.1)	16 (19.5)
1–5 years	16 (16)	4 (25)	5 (31.3)	5 (31.3)	1 (6.3)	1 (6.3)	0
>6 years	2 (2)	0	1 (50)	0	0	1 (50)	0

#### Knowledge of malaria

Almost all FGD participants showed knowledge of malaria. Locally termed *“Aai moi kap”* (disease mosquito bite) or *“Aai singyet”* (disease chills), respondents often recalled headache, fever, chills, shivering, vomiting, body pain as symptoms. Some also mentioned vomiting, thirst, cough and cold, and diarrhea.

#### Asymptomatic malaria infection as a concept

The concept of asymptomatic infection was new to participants and only few agreed that a healthy person could have malaria parasites in his or her blood. However, respondents also suspected that they could perhaps have a type of *malaria*, which they associated with the nature of their work and the cleanliness of conditions in their village. When probed about whether these healthy-looking but infected people could be infectious, almost all participants agreed.

*I*: *How do you know if somebody has malaria?*

*FGD7S1*: *When we go to forest and mosquito bite us.*

*FGD7S2*: *If a person goes to forest and mosquito bites but we cannot know from the look.*

*FGD7S3*: *If you see the mosquito bite and if that person has chills that means he/she has got malaria*

[After a brief pause]

*I*: *Let’s continue. How do you know if someone has malaria?*

*FGD7S5*: *By checking temperature*

*FGD7S6*: *By mosquito bite.*

*FGD7S7*: *if somebody takes medicine and got cured, we can know that person has malaria.*

*FGD7S8*: *We know it if somebody goes to hospital and finds out that he/she has malaria.*

FGD with eight female participants at a sub-village of TT

*I*: *Do you think that a healthy person (who is walking around and working normally) can be infected?*

*FGD6S1 (after discussing amongst themselves first)*: *All of us have malaria parasites inside our body because we are thin, we do not live clean, we always go to the forest and mosquito bites us. But for your project [TME] members, you do not have malaria parasite in your body because your lifestyle and living is the opposite to ours.*

FGD with 10 male participants at PMM

#### Malaria as a health problem in the villages

Many respondents described malaria as a major problem in their village. This was linked to their recognition of malaria as potentially fatal (if left untreated), past experiences when many community members died of the disease, or the out of pocket expenses for treatment, which can be a major burden for households. Respondents described sleeping under a mosquito net as essential to protect from the malaria and that inadequate number of mosquito nets prompted concern about this disease. Respondents also explained the presence of malaria in their villages in terms of their living conditions, particularly a reported lack of cleanliness, and the nature of their work in the forests, where they often receive mosquito bites.

*I*: *Is malaria a big problem in your village and if so why?*

*FGD6S1*: *Yes. It is big because a lot of people had malaria, many died and they had to spend a lot of money to treat it. (All other agreed).*

FGD with 10 male participants at PMM

*I*: *Malaria is a big problem in your village?*

*FGD5S2*: *Yes!*

*I*: *Why?*

*FGD5S2*: *We eat and stay unclean.*

*FGD5S8*: *Our clothes are unclean too, because we don’t have money to buy soap for washing clothes.*

*FGD5S7*: *Mosquito net is very old and broken.*

FGD with 8 female participants at PMM

#### Ways to eliminate malaria

The respondents gave multiple suggestions regarding how malaria could be eliminated: providing mosquito nets, wearing long sleeves, taking medicine when sick, cleaning the environment surrounding their homes, improving water and sanitation, and building health centers in their villages.

*I*: *Do you think malaria can be eliminated from the village? Why and How?*

*FGD12S2*: *Yes, we all the villagers have to sleep under the mosquito net. During the day, we have to wear long-sleeves.*

*FGD12S4*: *By using mosquito net.*

*FGD12S5*: *Medicine, mosquito net and toilet facility.*

*FGD12S6*: *Toilet facility*: *agreed with S5.*

FGD with 8 male participants at XT

*I*: *Do you think malaria can be eliminated from the village? Why and How?*

*FGD3S2*: *Yes [malaria can be eliminated], if the project helps.*

*FGD3S3*: *Yes, it is possible to eliminate if the project can build a health center here and check our health. And it should have enough doctors.*

*FGD3S8*: *By giving out mosquito nets.*

FGD with 8 female participants at sub-village of OTP

Taking an anti-malarial when healthy was a new idea. However, most respondents said that they would take it to eliminate malaria from the village and protect themselves, particularly because malaria was recognized as a deadly disease.

## Discussion

### Asymptomatic malaria infection as a concept

This is the first study to explore awareness of asymptomatic malaria infections in remote malaria-endemic communities in Laos. Asymptomatic malaria infection was a new concept and most respondents did not agree with the idea that a seemingly healthy person could have malaria parasites in his or her body. For those who were open to the concept of asymptomatic malaria, infection was often perceived to be associated with living conditions (lack of cleanliness and mosquito nets) and outdoor work (swidden farming and forest visits). Associating malaria with forest work and hygiene is not unique to Laos: research in sub-Saharan Africa and South East Asia has shown that community members often associate the risk of getting malaria from work in the forest and with poor hygiene [21, 42–44]. This is reflected in epidemiological research that has associated working in the forest and residing near the forest fringes with *Plasmodium* infections [45, 46].

Difficulties with grasping the idea of asymptomatic malaria infection have been reported elsewhere. In Cambodia even after intense community engagement accompanying MDA, community members and some study staff members struggled with the concept of asymptomatic *Plasmodium* infections [21]. Partial understanding, however, did not prevent community members from participating in MDA in Cambodia, Laos, or other sites [6, 20–24]. A multitude of factors embedded in local social and cultural context, community engagement, provision of health care and both monetary and non-monetary incentives have been found to affect the participation in Laos and elsewhere [6, 20, 21, 23, 24].

### Malaria as a health problem in the villages

In southern Savannakhet, as in western Cambodia, past experiences of malaria-related morbidity, mortality and treatment costs meant that malaria was described as a major health problem [21]. Malaria as a health concern was one of the factors that motivate community members to take part in subsequent pilot studies of MDA in these villages [24]. In addition, members of these remote communities often have little access to healthcare because of a lack of local facilities, poverty and poor transport infrastructure [6, 20, 24]. Thus constrained by the limited health care infra-structure and the economic burden that malaria entailed, respondents were further motivated by the perceived/real health benefits directly linked to MDA and the ancillary care TME provided [20, 24].

### Ways to eliminate/prevent malaria

Conventional malaria prevention measures–using mosquito nets, wearing long sleeves, treatment with anti-malarials when sick–were well known to respondents and were consistent with the findings of other studies [31–33]. Unsurprisingly, respondents were unaware of MDA as a potential malaria prevention strategy but showed enthusiasm towards participating in future MDAs. Intention to participate and enthusiasm to volunteer in MDA was also found amongst respondents in Cambodia [41]. In Laos, this was also reflected by a relatively high participation rate in subsequent MDA (>87% participated in three rounds of MDA) [6, 20, 24]. Participation in MDA was associated with attending study meetings, awareness of the study aim and the perception that MDA was worthwhile. Decisions about participation were also influenced by other household and community members [20]. Often community members were motivated to participate in the MDAs because of the perceived potential health benefits rather than understanding the scientific concept and rationale of MDA [20, 24].

Mass drug administrations were accompanied by intensive community engagement. This entailed informing and involving stakeholders and authority figures from central to the local level; local volunteers who were recruited and trained, and were integral to the design and implementation of activities; formative research to rapidly gain insight into the local social and cultural context, responsiveness whereby the approach was adapted according to the needs of the community and sharing control/leadership with the community in designing and executing program activities [6]. These elements were key in executing activities such as meetings, design of health education tools and house to house visits and aimed primarily to promote uptake of MDA [6, 20, 24].

### Implications for malaria control programs

Malaria control programs in the region are dependent on the peripheral health system and may not be able to undertake such intense community engagement and sensitization as in the pilot study that followed this research [6]. Programs however should incorporate the active collaboration of stakeholders, including policy makers, local health care workers, traditional healers and community members. This entails building a collaborative partnership with communities wherein members of the target populations can be trained to form a community malaria task force. Sharing responsibilities with community members can garner the “sense of ownership” and sustain malaria control and elimination efforts. A collaborative approach is likely to require more preparatory work, however, it was found economical compared to the vertical disease control programs in Africa [47, 48]. Such methods have been increasingly advocated and were found to be economical and sustainable in disease control programs [47–49].

### Strengths and limitations

Data were collected as part of formative research to inform subsequent community engagement for a TME pilot study in Laos. As a part of formative research, multiple questions on related theme were asked to explore how their responses varied, this enabled us to understand in-depth perceptions, knowledge, practice and attitudes. Three researchers collected data to reduce the possibility of personal bias caused by the influence of a single data collector. Cultural modesty and conformism including social desirability may have biased some of the data. Although using quantitative data analysis including logistic regression helped to consolidate the findings, the magnitude and accuracy of such data are limited by the lack of ability to explain the variable in detail. Nevertheless, incorporating a mixed method approach (quantitative and qualitative methods) enabled to elicit information from all households including the exploration of reasons behind particular responses (variables). This study derived the data from on-site translation from bilingual translators who were fluent in *Lao* and local language. The language spoken locally has no written script and some nuances and meaning could have been lost during translation. Care was therefore taken to involve several bi-lingual community members to whom study staff could seek clarification about local terminology.

It is possible that village heads who had previously been involved in sensitization meetings regarding the pilot study conducted by higher authorities beforehand had disseminated information on MDA and its rationale to fellow community members, which could have affected some of our results.

## Conclusion

In remote areas of Laos, prior to sensitization, awareness of asymptomatic malaria infections and MDA as a tool to eliminate malaria was low. This reflects attitudes to asymptomatic, *P*. *falciparum* infection elsewhere in the GMS. Nonetheless, malaria remains a major health concern and community members can generally describe its signs, symptoms and transmission. In the context of declining clinical *falciparum* and the higher proportion of *vivax* malaria, maintaining enthusiasm to clear asymptomatic *falciparum* parasite reservoir presents a challenge. Malaria control programs should invest in community engagement that, as well as seeking to inform people about asymptomatic infection, builds trust through the active collaboration of government stakeholders, key community members (community health workers, traditional healers and local leaders) and the wider community. This entails training and devolving responsibilities to the community members to sustain the control and elimination efforts.

## Supporting information

S1 Questionnaire Asymptomatic malaria infections. (PDF)

S1 Tables Additional analysis. (DOCX)
