# Supplementary material for: Perceptions of asymptomatic malaria infection and their implications for malaria control and elimination in Laos
Source: PLoS One. 2018 Dec 11;13(12):e0208912. doi: 10.1371/journal.pone.0208912 (PMC6289463; doi:10.1371/journal.pone.0208912)
Supplement: S1 Questionnaire — (PDF) [file pone.0208912.s001.pdf]

Targeted Malaria Elimination to eradicate malaria in areas of suspected or proven artemisinin resistance in Southeast Asia

BASELINE SURVEY: KNOWLEDGE, PECEPTIONS, ATTITUDE TOWARDS MALARIA AND MDA

INTRODUCTION

My name is………………… I am a doctor/social scientist working for a research team based in Mahosot hospital in Vientiane. We are doing health research in this village as part of the project called Target Malaria Elimination in Nong District, Savannakhet Province, Laos. We are trying to learn about the knowledge, attitudes and practices of villagers in relation to malaria and the targeted malaria elimination using mass administration of antimalarials. This will help us to improve the malaria control programme in the future. We would like to invite you to participate in our interview and would like to ask you some questions about malaria and other issues related to malaria. The interview will take about 30 minutes. You can refuse to participate in the interview at anytime. You can also refuse to answer some questions if you feel uncomfortable or you can stop giving answers at anytime – it is your right to refuse or to participate. We will respect your refusal to participate and will not continue to ask. Your refusal will not prevent you from having normal health service or other benefits and from taking part in normal activities within the village.

The participant gives consent to be interviewed □ (Continue to ask questions)

The participant does not give consent to be interviewed □ (Please fill information in Part I and stop asking further questions)

PART I: INITIALS

**Date of interview (DD/MM/YY)**	⎸_⎸_⎸/⎸_⎸_⎸/⎸_⎸_⎸_⎸_⎸
**Initials of Interviewer (First Name)**	⎸_⎸_⎸
**Language of Interview**	1 Laos<br>3 Local (Specify…………)

Targeted Malaria Elimination to eradicate malaria in areas of suspected or proven artemisinin resistance in Southeast Asia

**Household ID**	|_|_|_|_|_|_|_|_|_|_|
**Village Name or Code**	

PART II: SOCIODEMOGRAPHIC CHARACTERISTICS OF RESPONDENTS

1.	Respondent status in the family	
		1. Family Head
		2. Wife of Family Head
		3. Family Seniority (Specify………….)
		4. Other (specify)……………
2.	Age (years)	……………………..
3.	Sex	Male ☐ Female ☐ Other ☐
4.	Ethnicity	
		1. Laoloum (Specify…………..)
		2. Laothung (Specify…………..)
		3. Laosung (Specify…………..)
		4. Others (Specify…………..)
5.	What is your religion?	
		1. Buddhist
		2. Christian
		3. Animist
		4. Other: (Specify………………..)
6.	Marital status	
		1. Single
		2. Married
		3. Widow/widower
		4. Divorced/Separated
		5. Other (Specify…………………)
7.	Can you read and write Lao?	
		1. Yes, I can read and write
		2. I can read but cannot write

Targeted Malaria Elimination to eradicate malaria in areas of suspected or proven artemisinin resistance in Southeast Asia

		3. No, I cannot read nor write
8.	Which level did you finish your normal education?	*Please fill years of education as 0, if not attended at all*
		1. Years of education…………………..(years)
9.	Occupation	
		1. Farmer
		2. Student
		3. Trader/Business
		4. Civil servant (Specify……………)
		5. Labor/Manual worker
		6. Unemployed
		7. Driver
		8. Retired
		9. Other: Specify…………………
10.	How much income does your family make a month?	
		1. ≤500,000
		2. 500,001 to 1,000,000
		3. 1,000,001 to 2,000,000
		4. 2,000,001 to 3,000,000
		5. ≥3,000,001
		6. Don’t know
11.	What is your family daily expense?	……………………..Kip/day
12.	Is the amount of income enough to sustain living?	
		1. Yes
		2. No
		3. Not sure or do not know
13.	What do you own?	*(multiple options possible)*
		1.House
		2.Land for farming
		3.Motorbikes
		4.Bicycles
		5.Tractor

Targeted Malaria Elimination to eradicate malaria in areas of suspected or proven artemisinin resistance in Southeast Asia

		6.Cars
		7.Cattles
		8.Televisions
		9.Radio
		10.Mobile phones
		11.Generators
14.	What are the walls of your home made from? (Please tick it based on your observation)	
		Multiple options are possible
		1. Bamboo or grass or leaves
		2. Wood
		3. Mud
		4. Concrete
		5. Brick
		6. Plastic Sheeting
		7. Metal or CI sheet
		8. Other (Specify……………..)
		9. Don't know
15.	What is the roof of your home made from? (Please tick it based on your observation)	
		Multiple options are possible
		1. Bamboo or grass or leaves
		2. Wood
		3. Mud
		4. Concrete
		5. Brick
		6. Plastic Sheeting
		7. Metal or CI sheet
		8. Shingles
		9. Other (Specify……………..)
		10. Don't know
16.	What is the floor of your home made from? (Please tick it based on your observation)	
		Multiple options are possible
		1. Bamboo or grass or leaves
		2. Wood

Targeted Malaria Elimination to eradicate malaria in areas of suspected or proven artemisinin resistance in Southeast Asia

		3. Mud or soil
		4. Concrete/cement
		5. Brick
		6. Plastic Sheeting
		7. Metal
		8. Tiles
		9. Other (Specify……………..)
		10. Don't know
17.	Do you have toilet facility at home?	
		1.Yes
		2.No
17.1	*If No,* How or where do you defecate?	*(multiple options possible)*
		1. Backyard
		2. In the field
		3. In the forest
		4. In the river or stream
		5. Other (Specify…………………..)
17.2	*If Yes,* What kind of toilet facility do you have?	*(multiple options possible)*
		1. Flush toilet
		2. Pit latrine
		3. Vault toilet
		4. Other (Specify…………….)
		5. Don't know
18.	Did you migrate from any other village?	
		1. Yes
		2. No
18.1	*If Yes,* How long have you been living here?	
		1.……………… Years
19.	How far is the forest from your house?	
		1………………. Km

Targeted Malaria Elimination to eradicate malaria in areas of suspected or proven artemisinin resistance in Southeast Asia

		2……………….Mins
20.	How far is your rice field or plantation farm from your house?	
		1……………….Km
		2. ………………Min
		3. I don't have land
21.	How often do you go to forest?	
		1. Every day
		2. Every alternate day
		3. Weekly
		4. Every 2 weeks
		5. Every month
		6. Could not specify
		7. Not at all

PART III: HEALTH CARE SEEKING BEHAVIOUR

(I would like to ask you about the illness and how you deal with it)

22.	Have you ever become sick or had fever in the past few months?	
		1. Yes
		2. No
		3. Don't know
22.1	If yes, did you seek treatment?	
		1. Yes
		2. No
		3. Don't know
22.2	*If yes,* Where did you first go for treatment, when you felt sick?	
		1. Self-medication with Lao-traditional medicine
		2. Witchcraft
		3. Local drug store
		4. Private Clinic

Targeted Malaria Elimination to eradicate malaria in areas of suspected or proven artemisinin resistance in Southeast Asia

		5. Nearest health center/health post
		6. Others (specify)…………….
		7. Don't know/I don't remember
23.	If you are sick, where do you usually go for treatment first?	
		1. Self-medication with Lao-traditional medicine
		2. Witchcraft
		3. Local drug store
		4. Private Clinic
		5. Nearest health center/health post
		6. Will get cured by itself
		7. Others (specify)…………….
24.	Which do you prefer more between (Read out the options)?	
		1. Lao-traditional medicine
		2. Western Medical facilities (health centers)
25.	How far is nearest health center from your home?	……………..Km
26.	How do you get to nearest health center?	
		1. Walk
		2. Bicycle
		3. Cart
		4. Motorcycles
		5. Tractor
		6. Car
		7. Other (Specify…………….)
27.	Is the road to nearest health center in good condition?	
		1. Yes
		2. No
		3. Don't know
28.	Are you satisfied with your nearest health center?	
		1. Yes
		2. No

Targeted Malaria Elimination to eradicate malaria in areas of suspected or proven artemisinin resistance in Southeast Asia

		3. Don't know
28.1	*If No, Why?*	*Multiple options possible*
		1. Lack of health staffs
		2. Lack of medicine
		3. Often does not get cured from their treatment
		4. It's far to go
		5. They don't have specialties.
		6. Due to impolite behavior of health staffs
		7. They close down after office hours/holidays
		8. Perceived uncleanliness at health center.
		9. Absence of health staff during their visit
		10. Others (specify)…………..
29.	Can you get medicine without health personnel's prescription?	
		1. Yes
		2. No
		3. Don't know
30.	Do you prefer to get medicine without prescription from health personnel?	
		1. Yes
		2. No
		3. Don't know
30.1	*If Yes*, why?	
		1. It's easy to get medicine
		2. I don't like to go health center
		3. It is faster
		4. It is cheaper
		5. Because doctor/clinicians do not prescribe medicine.
		6. Other (Specify………………)

PART IV: KNOWLEDGE, PRACTICES, PERCEPTIONS AND ATTITUDES TOWARDS MALARIA

31.	Have you heard of malaria before?	

Targeted Malaria Elimination to eradicate malaria in areas of suspected or proven artemisinin resistance in Southeast Asia

		1. Yes
		2. No
		3. Don't know
31.1	*If yes*, what are the symptoms of malaria?	*Multiple answers possible*
		1.Fever
		2.Headache
		3.Muscle pain
		4.Vomitting
		5.Chills/shivering
		6.Sweating
		7.Diarrohea
		8.Sore throat
		9.Jaundince
		10.Running nose
		11.Other (Specify…………)
		12.Don't know
31.2	*If Yes*, Where did you get information on malaria from?	*Multiple answers possible*
		1. Radio
		2. TV
		3. Newspaper
		4. From families and friends
		5. Village meetings
		6. Health workers
		7. Banners/Boards/Flyers/leaflets
		8. Village head
		9. Other (Specify………….)
		10. Don't know
32.	Where would you like to get health information from?	*Multiple answers possible*
		1. Radio
		2. TV
		3. Newspaper
		4. From families and friends
		5. Village meetings
		6. Health workers
		7. Banners/Boards/Flyers/leaflets
		8. Entertainment Shows

Targeted Malaria Elimination to eradicate malaria in areas of suspected or proven artemisinin resistance in Southeast Asia

		9. Other (Specify…………….)	
33.	How can we get malaria? (From)	*Multiple answers possible*	
		1. Air	
		2. Water	
		3. Soil	
		4. Forest	
		5. Germ	
		6.Worm	
		7. Mosquito bite	
		8. God/Spirit	
		9. Uncleaned surroundings	
		10. Don't know	
		11. Other. (Specify……………….)	
34.	How do you prevent mosquito bites?	At Home	At Forest/rice field/farm
		1. Mosquito net	1. Mosquito net
		2. Burning the wood	2. Burning the wood
		3. Using Hammock net	3. Using Hammock net
		4. Using repellants	4. Using repellants
		5. Smoking	5. Smoking
		6. Oral medicine	6. Oral medicine
		7. Wear sleeved clothes	7. Wear sleeved clothes
		8. Nothing	8. Nothing
		7.Other (Specify……….)	7.Other (Specify……….)
35.	Did you sleep under the mosquito net last night?		
		1. Yes	
		2. No	
35.1	*If Yes*, How often do you sleep under the mosquito net?		
		1.Everyday	
		2.Sometimes (>2-3 days/week)	
		3.Rarely (once in a while)	
36.	How many Mosquito nets do you have in your home?		
		1. (Number…………….)	

Targeted Malaria Elimination to eradicate malaria in areas of suspected or proven artemisinin resistance in Southeast Asia

		2. Don't know
37.	Do you have impregnated nets?	
		1. Yes
		2. No
		3. Don't know
38.	Is mosquito net available for each family member?	
		1. Yes
		2. No
		3.Don't know
39.	Do you think you can get malaria if anybody in your family or neighbor has malaria?	
		1. Yes
		2. No
		3. Don't know
39.1	*If Yes*, How? (Through)	*Multiple answers possible*
		1. Touch
		2. Air
		3. Mosquito bite
		4. Water
		5. Food
		6. Don't know
		7. Other (Specify………………..)
40.	How do we know if a person has malaria?	*Multiple answers possible*
		1. Through blood test
		2. That person will have fever, chills and headache
		3. Went to see health worker
		4. Went to the forest before
		5. Others (specify)……………..
41.	Is malaria a deadly disease?	
		1. Yes
		2. No
		3. Don't know

Targeted Malaria Elimination to eradicate malaria in areas of suspected or proven artemisinin resistance in Southeast Asia

42.	Are you scared of malaria?	
		1. Yes
		2. No
		3. Don't know
42.1	*If scared*, Why?	
		1. It can take life
		2. It causes poor health
		3. It causes extra expenses for the treatment
		4. I cannot work after I am sick
		5. Other (Specify…………..)
		6. Don't know
42.2	*If Not scared*, Why?	
		1. The treatment is free of charge
		2. I will get compensation.
		3.It doesn't affect me very much
		4. Easy to access the health center
		5. Don't know
		6. Other (Specify…………..)
43.	Do you think that a healthy person can still have malaria in his/her blood without feeling sick?	
		1. Yes
		2. No
		3. Don't know
43.1	*If Yes*, Is presence of malaria in blood dangerous?	
		1. Yes
		2. No
		3. Don't know
44.	Can malaria be cured by medicine?	
		1. Yes
		2. No
		3. Don't know

Targeted Malaria Elimination to eradicate malaria in areas of suspected or proven artemisinin resistance in Southeast Asia

44.1	*If Yes,* How many days should medicine be taken for treatment?	
		1. Days…………………
		2. Don't know
45.	If you feel better after day 1 or day 2, would you still take medicine for next few days to complete the course given by health worker?	
		1. Yes
		2. No
		3. Don't know
46.	Have you ever had malaria in your lifetime?	
		1.Yes
		2. No
		3. Don't know
46.1	*If yes,* when did you have the last malaria episode?	
		1. Few days ago
		2. Few weeks ago
		3. Few months ago
		4. More than a year ago
		5. I don't remember/Don't know
46.2	*If yes,* how did you know that you got malaria last time?	*Multiple answers possible*
		1. Blood test
		2. Health worker told me after blood testing
		3. Health worker told me without blood test
		4. I thought my illness looked like malaria
		5. I knew it from witchcraft/traditional healer
		6. Other (specify)…………….
46.3	*If Yes,* Where did you go for treatment when you got the last malaria episode?	*Multiple answers possible*
		1. Lao Traditional healer
		2. Witchcraft

Targeted Malaria Elimination to eradicate malaria in areas of suspected or proven artemisinin resistance in Southeast Asia

		3. District Hospital
		4. Local health center
		5. Drug store
		6. Self-medicated
		7. Other. Specify……………….
		8. I don't remember
46.4	*If Yes*, How many days did you take antimalarial for?	
		1. 1 day
		2. 2 days
		3. 3 days
		4. More than 3 days
		5. I did not take it
		6. Don't know/Don't remember
47.	Do you think health centers in your community are capable of treating malaria?	
		1. Yes
		2. No
		3. Don't know

PART V: KNOWLEDGE, PERCEPTIONS AND ATTITUDES TOWARDS MASS DRUG ADMINISTRATION

48.	Is malaria a big problem in your community?	
		1. Yes
		2. No
		3. Don't know
49.	Do you think a person in your village can have malaria parasite in his/her body without being sick?	
		1. Yes
		2. No
		3. Don't know
49.1	*If yes*, why are these people not sick?	*Multiple answers possible*
		1. They are healthy or strong

Targeted Malaria Elimination to eradicate malaria in areas of suspected or proven artemisinin resistance in Southeast Asia

		2. They have immunity
		3. They take malaria medicine all the time
		4. They have very little malaria parasites in their body
		5. Others (specify)……………..
		6. Don’t know
49.2	*If yes*, Are these people dangerous?	1. Yes
		2. No
		3. Don't Know
49.2.1	*If Yes*, Why are they dangerous?	*Multiple answers possible*
		1. They can transmit or give disease to others
		2. They can become sick one day in the future
		3. Others (specify)………..
		4. Don’t know
50.	Can a healthy person, who have malaria parasites in his/her body, transmit the disease to others?	
		1. Yes
		2. No
		3. Don't know
50.1	*If yes*, what should we do to these people?	
		1. Give them malaria medicine
		2. Do nothing
		3. Send them to health center
		4. Chase them away from village
		5. Don't know
		6. Other (Specify…………….)
50.2	*If Yes*, since we do not know who has malaria parasites in their bodies without illness, should we give malaria medicine to everyone in this village?	
		1. Yes

Targeted Malaria Elimination to eradicate malaria in areas of suspected or proven artemisinin resistance in Southeast Asia

		2. No
		3. Don't know
50.2.1	*If yes*, why?	*Multiple answers possible*
		1. To cure all people
		2. To eliminate malaria from village
		3.To prevent malaria transmission in the village
		4. To prevent us from malaria
		5. Don't know
		6. Others (specify)………….
50.2.2	*If no,* why?	*Multiple answers possible*
		1. It is dangerous for those who do not have malaria parasites in their bodies
		2. Malaria medicines are toxic
		3. People become sick with these medicines
		4. Don't know
		5. Others (specify)…………
51.	Have you heard of current malaria elimination in your village?	
		1. Yes
		2. No
		3. Don't know
52.	Do you think malaria can be eliminated from your community?	
		1. Yes
		2. No
		3. Don't know
52.1	*If Yes,* How?	*Multiple answers possible*
		1.By giving medicines to all the people in the village
		2. By using mosquito nets
		3. By taking regular medicine
		4. By cleaning the surrounding
		5. By using traditional Lao medicine
		6.Witchcraft
		8. Don't know

Targeted Malaria Elimination to eradicate malaria in areas of suspected or proven artemisinin resistance in Southeast Asia

		7. Other (Specify…………….)
52.2	*Should* entire community be involved to eliminate malaria?	
		1. Yes
		2. No
		3. Don’t know
53.	Would you participate in malaria elimination as a volunteer?	
		1. Yes
		2. No
		3. Don’t know
53.1	*If Yes*, why?	
		1. I want to make my community free from malaria
		2. I want to help my community
		3. Malaria is a big problem in my community
		4. Other (Specify………………)
		5. Don’t know
53.2	*If No*, Why?	
		1. I have no time or I am busy
		2. I haven’t understood about this program
		3. I don’t see malaria as a big problem
		4. Other (Specify…………….)
		5. Don’t know
54.	To eliminate a disease from a community, one way is to give medicines to all people regardless of whether they have the disease or not (this is called MDA), have you ever heard about MDA before?	
		1. Yes
		2. No
		3. Don’t know
54.1	*If yes*, where did you hear this from?	
		1. Health personnel

Targeted Malaria Elimination to eradicate malaria in areas of suspected or proven artemisinin resistance in Southeast Asia

		2. Friends/family/villagers
		3. Radio/TV/Newspaper
		4. Village head
		5. Other (Specify…………)
54.2	*If yes*, have you ever taken part in any MDA before?	
		1. Yes
		2. No
		3. Don't know
54.3	*If yes*, Where and when did you take part in MDA?	
		1. District…………….2. Year…………..
55.	To eliminate malaria from a community, one way is to give malaria medicines to all people regardless of whether they have malaria parasites in their bodies or not (this is called malaria MDA), do you agree with this idea?	
		1. Yes
		2. No
		3. Don't know
55.1	*If Yes*, Why?	
		1. It protects us from malaria
		2. I want community to be free from malaria
		3. Specify (………………………..)
55.2	*If No*, Why?	
		1. I am scared of medicine
		2. I don't want to take medicine when I am not sick
		3. Specify (…………………………)
56.	If MDA is going to happen in this village, all people's blood should be	

Targeted Malaria Elimination to eradicate malaria in areas of suspected or proven artemisinin resistance in Southeast Asia

	tested and take medicine for malaria, would you take part in this MDA?	
		1. Yes
		2. No
		3. Don’t know
56.1	*If Yes,* Why?	
		1. I want my community to be free from malaria
		2. I am not scared of it
		3. I want to know if there are malaria parasites in my body
		4. Specify (……………………………..)
56.2	*If No,* Why?	
		1. The medicine for MDA is dangerous
		2. I am scared of needle
		3. Specify (…………………………)
57.	Are you scared to give blood for check-up?	
		1. Yes
		2. No
		3. Don’t know
57.1	*If Yes,* why?	*Multiple answers possible*
		1. We lose energy
		2. It is painful
		3. It can bring other diseases
		4. We run out of blood
		5. Don’t know
		6. People perceive that they sell blood
		7. Scared of needle
		8. Other (Specify…………)
58.	Would you take medicine for mass drug administration?	
		1. Yes
		2. No
		3. Don’t know
58.1	*If No,* why?	
		1. I only take medicine when I am sick

Targeted Malaria Elimination to eradicate malaria in areas of suspected or proven artemisinin resistance in Southeast Asia

		2. I am scared of medicine side effects
		3. I do not trust these medicines
		4. I do not like to take medicine
		5. Other (Specify………..)
		6. Don’t know

Thank you very much!
