# Supplementary material for: Perceptions of asymptomatic malaria infection and their implications for malaria control and elimination in Laos
Source: PLoS One. 2018 Dec 11;13(12):e0208912. doi: 10.1371/journal.pone.0208912 (PMC6289463; doi:10.1371/journal.pone.0208912)
Supplement: S1 Tables — (DOCX) [file pone.0208912.s002.docx]

**S1 Appendix:** Additional analysis (Tables A1 to A4)

| **Table A1: Socio-demographic characteristics of participants in relation to perception on asymptomatic malaria infections (n=281)** | | | | | |
| --- | --- | --- | --- | --- | --- |
|  | **Total** | **Possibility of asymptomatic malaria infections** | | | **p-value** |
| **Characteristics** | **Number (%)** | **Yes (n=40)** | **No (n=146)** | **Don’t know (n=95)** |  |
| **Village** |  |  |  |  |  |
| OTP | 65 (23.1) | 16 (40) | 22 (15.1) | 27 (28.4) | **<0.001** |
| PMM | 74 (26.3) | 2 (5) | 48 (32.9) | 24 (25.3) |  |
| TT | 82 (29.2) | 5 (12.5) | 43 (29.5) | 34 (35.8) |  |
| XT | 60 (21.4) | 17 (42.5) | 33 (22.6) | 10 (10.5) |  |
| **Respondent status** | |  |  |  |  |
| Family head | 188 (66.9) | 29 (72.5) | 103 (70.5) | 56 (58.9) | **0.009** |
| Wife of family head | 70 (24.9) | 10 (25) | 26 (17.8) | 34 (35.8) |  |
| Other | 23 (8.2) | 1 (2.5) | 17 (11.6) | 5 (5.3) |  |
| **Age Group** |  |  |  |  |  |
| ≤30 years | 91 (32.4) | 13 (32.5) | 51 (34.9) | 27 (28.4) | 0.31 |
| 31-50 years | 138 (49.1) | 23 (57.5) | 64 (43.8) | 51 (53.7) |  |
| ≥51 years | 52 (18.5) | 4 (10) | 31 (21.2) | 17 (17.9) |  |
| Mean=38.9±14.5, min=18 and max=100 | | | | | |
| **Sex** |  |  |  |  |  |
| Male | 201 (71.5) | 30 (75) | 113 (77.4) | 58 (61.1) | **0.02** |
| Female | 80 (28.5) | 10 (25) | 33 (22.6) | 37 (38.9) |  |
| **Ethnicity*** |  |  |  |  |  |
| Lao Theung | 269 (95.7) | 32 (80) | 144 (98.6) | 93 (97.9) | **<0.001** |
| Other | 12 (4.3) | 8 (20) | 2 (1.4) | 2 (2.1) |  |
| **Religion** |  |  |  |  |  |
| Buddhist | 9 (3.2) | 8 (20) | 1 (0.7) | 0 | **<0.001** |
| Animist | 272 (96.8) | 32 (80) | 145 (99.3) | 95 (100) |  |
| **Marital Status** |  |  |  |  |  |
| In relationship | 262 (93.2) | 39 (97.5) | 132 (90.4) | 91 (95.8) | 0.13 |
| Not in relationship | 19 (6.8) | 1 (2.5) | 14 (9.6) | 4 (4.2) |  |
| **Literacy** |  |  |  |  |  |
| Literate | 67 (23.8) | 18 (45) | 37 (25.3) | 12 (12.6) | **<0.001** |
| Illiterate | 214 (76.2) | 22 (55) | 109 (74.7) | 83 (87.4) |  |
| **Education in years** | |  |  |  |  |
| Not attended School | 204 (72.6) | 22 (55) | 101 (69.2) | 81 (85.3) | **0.001** |
| Attended School | 77 (27.4) | 18 (45) | 45 (30.8) | 14 (14.7) |  |
| **Occupation** |  |  |  |  |  |
| Farmer | 254 (90.4) | 29 (72.5) | 137 (93.8) | 88 (92.6) | **<0.001** |
| Other | 27 (9.6) | 11 (27.5) | 9 (6.2) | 7 (7.4) |  |
| **Monthly Income** | |  |  |  |  |
| ≤500,000 kip | 192 (68.3) | 14 (35) | 117 (80.1) | 61 (64.2) | **<0.001** |
| 500,001 to 2,000,000 kip | 41 (14.6) | 11 (27.5) | 15 (10.3) | 15 (15.8) |  |
| ≥ 2000,001 | 27 (9.6) | 11 (27.5) | 6 (4.1) | 10 (10.5) |  |
| Don't know | 21 (7.5) | 4 (10) | 8 (5.5) | 9 (9.5) |  |
| **Do you have toilet facility at home?** | | |  |  |  |
| Yes | 22 (7.8) | 4 (10) | 13 (8.9) | 5 (5.3) | 0.5 |
| No | 259 (92.2) | 36 (90) | 133 (91.1) | 90 (94.7) |  |
| **Did you migrate from any other village?** | | |  |  |  |
| Yes | 61 (21.7) | 13 (32.5) | 30 (20.5) | 18 (18.9) | 0.19 |
| No | 220 (78.3) | 27 (67.5) | 116 (79.5) | 77 (81.1) |  |
| **How far is the forest from your house in km?** | | | |  |  |
| ≤1 km | 97 (34.5) | 10 (25) | 53 (36.3) | 34 (35.8) | **0.006** |
| 1.1 to 2 km | 76 (27) | 13 (32.5) | 42 (28.8) | 21 (22.1) |  |
| ≥2.1 km | 49 (17.4) | 5 (12.5) | 33 (22.6) | 11 (11.6) |  |
| NA | 59 (21) | 12 (30) | 18 (12.3) | 29 (30.5) |  |
| **How often do you go to forest?** | | |  |  |  |
| Everyday | 171 (60.9) | 24 (60) | 93 (63.7) | 54 (56.8) | 0.19 |
| Every alternate day | 68 (24.2) | 7 (17.5) | 38 (26) | 23 (24.2) |  |
| ≥Weekly | 42 (14.9) | 9 (22.5) | 15 (10.3) | 18 (18.9) |  |
| ***Ka Tarng=1 (0.4%), Lao Loum=3 (1.1%), Mangkong=200 (71.2), Phu Thai=6 (2.1%), Ta Oi=3 (1.1%), Tree=64 (22.8%), Vietnamese=4 (1.4%)** | | | | | |

| **Table A2:Knowledge, practices, perceptions and attitudes towards malaria of participants in relation to perception on asymptomatic malaria infections (n=281)** | | | | | |
| --- | --- | --- | --- | --- | --- |
|  | **Total** | **Possibility of asymptomatic malaria infections** | | | **p-value** |
| **Characteristics** | **Number (%)** | **Yes (n=40)** | **No (n=146)** | **Don’t know (n=95)** |  |
| **Have you heard of malaria before?** | | |  |  |  |
| Yes | 260 (92.5) | 38 (95) | 135 (92.5) | 87 (91.6) | 0.77 |
| No | 18 (6.4) | 2 (5) | 10 (6.8) | 6 (6.3) |  |
| Don't know | 3 (1.1) | 0 | 1 (0.7) | 2 (2.1) |  |
| **What are the symptoms of malaria? (n=260)*** | | | |  |  |
| Fever | 231 (88.8) | 33 (86.8) | 124 (91.9) | 74 (85.1) | 0.26 |
| Headache | 190 (73.1) | 32 (84.2) | 107 (79.3) | 51 (58.6) | **0.001** |
| Muscle pain | 46 (17.7) | 12 (31.6) | 25 (18.5) | 9 (10.3) | **0.016** |
| Vomiting | 45 (17.3) | 7 (18.4) | 29 (21.5) | 9 (10.3) | 0.09 |
| Chills | 227 (87.3) | 32 (84.2) | 119 (88.1) | 76 (87.4) | 0.81 |
| **Where did you get the information on malaria from? (n=260)*** | | | | |  |
| Village Meetings | 3 (1.2) | 1 (2.6) | 2 (1.5) | 0 | 0.39 |
| Health workers | 193 (74.2) | 24 (63.2) | 105 (77.8) | 64 (73.6) | 0.18 |
| Banners | 2 (0.8) | 2 (5.3) | 0 | 0 | **0.003** |
| Other | 49 (18.8) | 8 (21.1) | 20 (14.8) | 21 (24.1) | 0.2 |
| Don’t know | 41 (15.8) | 13 (34.2) | 19 (14.1) | 9 (10.3) | **0.003** |
| **Where would you like to get health information from?*** | | | |  |  |
| Village Meetings | 59 (21) | 9 (22.5) | 40 (27.4) | 10 (10.5) | **0.007** |
| Health workers | 137 (48.8) | 23 (57.5) | 59 (40.4) | 55 (57.9) | **0.014** |
| Banners | 56 (19.9) | 6 (15) | 39 (26.7) | 11 (11.6) | **0.011** |
| Entertainment | 52 (18.5) | 9 (22.5) | 18 (12.3) | 25 (26.3) | **0.019** |
| Other | 13 (4.6) | 2 (5) | 8 (5.5) | 3 (3.2) | 0.69 |
| Don’t know | 31 (11) | 5 (12.5) | 11 (7.5) | 15 (15.8) | 0.12 |
| **Malaria is transmitted from*** | |  |  |  |  |
| Water | 14 (5) | 2 (5) | 10 (6.8) | 2 (2.1) | 0.25 |
| Soil | 1 (0.4) | 1 (2.5) | 0 | 0 | **0.04** |
| Forest | 55 (19.6) | 9 (22.5) | 34 (23.3) | 12 (12.6) | 0.11 |
| Mosquito | 230 (81.9) | 37 (92.5) | 125 (85.6) | 68 (71.6) | **0.004** |
| God | 1 (0.4) | 0 | 1 (0.7) | 0 | 0.62 |
| Uncleaned Surrounding | 30 (10.7) | 4 (10) | 19 (13) | 7 (7.4) | 0.37 |
| Don’t know | 29 (10.3) | 0 | 9 (6.2) | 20 (21.1) | **<0.001** |
| **How do you prevent mosquito bites at home?*** | | | |  |  |
| Using mosquito net | 274 (97.5) | 40 (100) | 144 (98.6) | 90 (94.7) | 0.09 |
| Using repellants | 5 (1.8) | 1 (2.5) | 2 (1.4) | 2 (2.1) | 0.85 |
| By smoking | 2 (0.7) | 0 | 2 (1.4) | 0 | 0.39 |
| Wearing Sleeves | 16 (5.7) | 3 (7.5) | 6 (4.1) | 7 (7.4) | 0.49 |
| **How do you prevent mosquito bites at forest?*** | | | |  |  |
| Using mosquito net | 1 (0.4) | 0 | 0 | 1 (1.1) | 0.37 |
| Burning fire | 11 (3.9) | 2 (5) | 7 (4.8) | 2 (2.1) | 0.53 |
| Using repellants | 7 (2.5) | 5 (12.5) | 1 (0.7) | 1 (1.1) | **<0.001** |
| By smoking | 1 (0.4) | 1 (2.5) | 0 | 0 | **0.049** |
| Wearing Sleeves | 262 (93.2) | 39 (97.5) | 135 (92.5) | 88 (92.6) | 0.5 |
| *Multiple answers were possible and percentage does not add to 100, analyses were made against "yes" and "no". | | | | | |

| **Table A3:Knowledge, practices, perceptions and attitudes towards malaria of participants in relation to perception on asymptomatic malaria infections (n=281)** | | | | | |
| --- | --- | --- | --- | --- | --- |
|  | **Total** | **Possibility of asymptomatic malaria infections** | | | **p-value** |
| **Characteristics** | **Number (%)** | **Yes (n=40)** | **No (n=146)** | **Don’t know (n=95)** |  |
| **Did you sleep under the mosquito net last night?** | | | |  |  |
| Yes | 256 (91.1) | 38 (95) | 135 (92.5) | 83 (87.4) | 0.25 |
| No | 25 (8.9) | 2 (5) | 11 (7.5) | 12 (12.6) |  |
| **If Yes, how often do you sleep under the mosquito net?** | | | |  |  |
| Everyday | 247 (96.5) | 35 (92.1) | 132 (97.8) | 80 (96.4) | 0.24 |
| Sometimes (>2-3 days in a week) | 9 (3.5) | 3 (7.9) | 3 (2.2) | 3 (3.6) |  |
| **How many mosquito nets do you have at your home?** | | | |  |  |
| ≤2 | 156 (55.5) | 23 (57.5) | 84 (57.5) | 49 (51.6) | 0.63 |
| ≥3 | 125 (44.5) | 17 (42.5) | 62 (42.5) | 46 (48.4) |  |
| **Do you think you can get malaria if anybody in your family/neighbor has malaria?** | | | | | |
| Yes | 164 (58.4) | 27 (67.5) | 92 (63) | 45 (47.4) | **<0.001** |
| No | 83 (29.5) | 11 (27.5) | 47 (32.2) | 25 (26.3) |  |
| Don't know | 34 (12.1) | 2 (5) | 7 (4.8) | 25 (26.3) |  |
| **If Yes, how? (n=164)*** | |  |  |  |  |
| Touch | 75 (45.7) | 8 (29.6) | 52 (56.5) | 15 (33.3) | **0.007** |
| Air | 11 (6.7) | 0 | 10 (10.9) | 1 (2.2) | 0.051 |
| Mosquito | 108 (65.9) | 21 (77.8) | 60 (65.2) | 27 (60) | 0.3 |
| Water | 33 (20.1) | 4 (14.8) | 26 (28.3) | 3 (6.7) | **0.009** |
| Food | 34 (20.7) | 4 (14.8) | 25 (27.2) | 5 (11.1) | 0.06 |
| **How do we know if a person has malaria?*** | | |  |  |  |
| Blood | 178 (63.3) | 30 (75) | 89 (61) | 59 (62.1) | 0.25 |
| Fever | 73 (26) | 14 (35) | 39 (26.7) | 20 (21.1) | 0.23 |
| Health worker | 90 (32) | 11 (27.5) | 64 (43.8) | 15 (15.8) | **<0.001** |
| Other | 33 (11.7) | 2 (5) | 18 (12.3) | 13 (13.7) | 0.34 |
| **Is malaria a deadly disease?** | |  |  |  |  |
| Yes | 265 (94.3) | 40 (100) | 141 (96.6) | 84 (88.4) | **0.014** |
| No | 12 (4.3) | 0 | 5 (3.4) | 7 (7.4) |  |
| Don't know | 4 (1.4) | 0 | 0 | 4 (4.2) |  |
| **Are you scared of malaria?** | |  |  |  |  |
| Yes | 277 (98.6) | 39 (97.5) | 145 (99.3) | 93 (97.9) | 0.56 |
| No | 3 (1.1) | 1 (2.5) | 1 (0.7) | 1 (1.1) |  |
| Don't know | 1 (0.4) | 0 | 0 | 1 (1.1) |  |
| **Can malaria be cured by medicine?** | | |  |  |  |
| Yes | 275 (97.9) | 38 (95) | 144 (98.6) | 93 (97.9) | 0.26 |
| No | 3 (1.1) | 1 (2.5) | 2 (1.4) | 0 |  |
| Don't know | 3 (1.1) | 1 (2.5) | 0 | 2 (2.1) |  |
| *Multiple answers were possible and percentage does not add to 100, analyses were made against "yes" and "no". | | | | | |

| **Table A4:Knowledge, perceptions and attitudes towards MDA of participants in relation to perception on asymptomatic malaria infections (n=281)** | | | | | |
| --- | --- | --- | --- | --- | --- |
|  | **Total** | **Possibility of asymptomatic malaria infections** | | | **p-value** |
| **Characteristics** | **Number (%)** | **Yes (n=40)** | **No (n=146)** | **Don’t know (n=95)** |  |
| **Is malaria a big problem in your community?** | | |  |  |  |
| Yes | 178 (63.3) | 29 (72.5) | 98 (67.1) | 51 (53.7) | **0.005** |
| No | 43 (15.3) | 8 (20) | 23 (15.8) | 12 (12.6) |  |
| Don't know | 60 (21.4) | 3 (7.5) | 25 (17.1) | 32 (33.7) |  |
| **Do you think a person in your village can have malaria parasite without being sick?** | | | | | |
| Yes | 34 (12.1) | 26 (65) | 6 (4.1) | 2 (2.1) | **<0.001** |
| No | 144 (51.2) | 9 (22.5) | 119 (81.5) | 16 (16.8) |  |
| Don't know | 103 (36.7) | 5 (12.5) | 21 (14.4) | 77 (81.1) |  |
| **Can a healthy person with malaria parasite in his body transmit to others?** | | | | |  |
| Yes | 103 (36.7) | 23 (57.5) | 53 (36.3) | 27 (28.4) | **<0.001** |
| No | 94 (33.5) | 13 (32.5) | 68 (46.6) | 13 (13.7) |  |
| Don't know | 84 (29.9) | 4 (10) | 25 (17.1) | 55 (57.9) |  |
| **If Yes, should we provide medicine to all the villagers?** | | | |  |  |
| Yes | 78 (75.7) | 17 (73.9) | 41 (77.4) | 20 (74.1) | 0.45 |
| No | 19 (18.4) | 6 (26.1) | 9 (17) | 4 (14.8) |  |
| Don't know | 6 (5.8) | 0 | 3 (5.7) | 3 (11.1) |  |
| **If Yes, why? (n=78)*** | |  |  |  |  |
| To cure all villagers | 29 (37.2) | 11 (64.7) | 15 (36.6) | 3 (15) | **0.008** |
| To eliminate malaria from the village | 15 (19.2) | 4 (23.5) | 8 (19.5) | 3 (15) | 0.8 |
| To prevent malaria transmission in the village | 41 (52.6) | 9 (52.9) | 18 (43.9) | 14 (70) | 0.15 |
| To prevent us from malaria | 34 (43.6) | 7 (41.2) | 13 (31.7) | 14 (70) | **0.018** |
| **Have you heard of malaria elimination in your village?** | | | |  |  |
| Yes | 40 (14.2) | 8 (20) | 19 (13) | 13 (13.7) | **0.046** |
| No | 224 (79.7) | 32 (80) | 121 (82.9) | 71 (74.7) |  |
| Don't know | 17 (6) | 0 | 6 (4.1) | 11 (11.6) |  |
| **Do you think malaria can be eliminated from your village?** | | | |  |  |
| Yes | 162 (57.7) | 30 (75) | 82 (56.2) | 50 (52.6) | **0.009** |
| No | 66 (23.5) | 7 (17.5) | 41 (28.1) | 18 (18.9) |  |
| Don't know | 53 (18.9) | 3 (7.5) | 23 (15.8) | 27 (28.4) |  |
| **If Yes, how? (n=162)*** | |  |  |  |  |
| By giving medicine to all villagers | 105 (64.8) | 24 (80) | 56 (68.3) | 25 (50) | **0.016** |
| By using mosquito net | 81 (50) | 17 (56.7) | 45 (54.9) | 19 (38) | 0.12 |
| By taking regular medicine | 9 (5.6) | 1 (3.3) | 7 (8.5) | 1 (2) | 0.23 |
| By cleaning surrounding | 23 (14.2) | 6 (20) | 13 (15.9) | 4 (8) | 0.27 |
| Don't know | 11 (6.8) | 1 (3.3) | 3 (3.7) | 7 (14) | **0.051** |
| Other | 30 (18.5) | 6 (20) | 8 (9.8) | 16 (32) | **0.006** |
| **Would you participate in malaria elimination as a volunteer?** | | | |  |  |
| Yes | 208 (74) | 35 (87.5) | 100 (68.5) | 73 (76.8) | **0.07** |
| No | 60 (21.4) | 5 (12.5) | 39 (26.7) | 16 (16.8) |  |
| Don't know | 13 (4.6) | 0 | 7 (4.8) | 6 (6.3) |  |
| **If Yes, why? (n=208)*** | |  |  |  |  |
| I want to make my community free from malaria | 127 (61.1) | 29 (82.9) | 53 (53) | 45 (61.6) | **0.008** |
| I want to help my community | 85 (40.9) | 14 (40) | 56 (56) | 15 (20.5) | **<0.001** |
| Malaria is a big problem in my community | 6 (2.9) | 2 (5.7) | 1 (1) | 3 (4.1) | 0.26 |
| Other | 14 (6.7) | 0 | 5 (5) | 9 (12.3) | **0.036** |
| Don't know | 21 (10.1) | 1 (2.9) | 6 (6) | 14 (19.2) | **0.005** |
| **Have your ever heard about MDA before?** | | |  |  |  |
| Yes | 257 (91.5) | 36 (90) | 128 (87.7) | 93 (97.9) | **0.028** |
| No | 16 (5.7) | 2 (5) | 14 (9.6) | 0 |  |
| Don't know | 8 (2.8) | 2 (5) | 4 (2.7) | 2 (2.1) |  |
| **Would you take part in MDA in future?** | | |  |  |  |
| Yes | 198 (70.5) | 39 (97.5) | 91 (62.3) | 68 (71.6) | **<0.001** |
| No | 82 (29.2) | 1 (2.5) | 55 (37.7) | 26 (27.4) |  |
| Don't know | 1 (0.4) | 0 | 0 | 1 (1.1) |  |
| *Multiple answers were possible and percentage does not add to 100, analyses were made against "yes" and "no". | | | | | |
